# Characterization of the immune microenvironment in brain metastases from different solid tumors

**DOI:** 10.1002/cam4.2905

**Published:** 2020-02-04

**Authors:** Jinling Jiang, Lihong Wu, Fei Yuan, Jun Ji, Xiaojing Lin, Wanning Yang, Junwei Wu, Min Shi, Hui Yang, Yanna Ma, Xue Song, Zhenggang Zhu, Henghui Zhang, Jun Zhang

**Affiliations:** ^1^ Department of Oncology Ruijin Hospital Shanghai Jiaotong University School of Medicine Shanghai China; ^2^ Genecast Precision Medicine Technology Institute Beijing China; ^3^ Department of Pathology Ruijin Hospital Shanghai Jiaotong University School of Medicine Shanghai China; ^4^ Shanghai Institute of Digestive Surgery Ruijin Hospital Shanghai Jiaotong University School of Medicine Shanghai China; ^5^ Institute of Infectious Diseases Beijing Ditan Hospital Capital Medical University Beijing China

**Keywords:** brain metastasis, immune checkpoint, immune system, lymphocyte, PD‐L1

## Abstract

**Background:**

Brain metastases are one of the most common intracranial neoplasms. Increasing evidence have indicated that systemic immunotherapy may provide long‐term benefits for brain metastases. Herein, we presented the results of an immune oncology panel RNA sequencing platform for patients with brain metastases from different primary sites.

**Methods:**

We investigated 25 samples of human brain metastases from lung cancer (n = 12), breast cancer (n = 6), and colorectal cancer (n = 7). Besides, 13 paired samples of adjacent noncancerous brain tissue (10 from patients with lung cancer and 3 from patients with breast cancer) were collected as controls. By comparing the brain metastases and paired samples of adjacent noncancerous brain tissue from 13 patients, we detected three upregulated and six downregulated genes, representing the malignant properties of cancer cells and increased immune infiltration in the microenvironment. Next, we profiled the immune‐related genes in brain metastases from three primary cancer types.

**Results:**

A group of genes were significantly overexpressed in the microenvironment of brain metastases from lung cancer, covering the checkpoint pathways, lymphocyte infiltration, and TCR‐coexpression. Especially, immune checkpoint molecules, PD‐L1, PD‐L2, and IDO1 were expressed at higher levels in brain metastases from lung cancer than those from the other two cancer types.

**Conclusions:**

This study presents an immune landscape of brain metastases from different cancer types. With high RNA expression levels of PD‐1/PD‐L1 axis and immune infiltration in brain metastases, it would be worthwhile to explore the efficacy of immune checkpoint blockade for lung cancer patients with intracranial metastases.

## Graphical abstract

Our data revealed that a group of genes were significantly overexpressed in the microenvironment of brain metastases from lung cancer, covering the checkpoint pathways, lymphocyte infiltration, and TCR‐coexpression. Especially, immune checkpoint molecules, PD‐L1, PD‐L2, and IDO1 were expressed at higher levels in brain metastases from lung cancer than those from the other two cancer types.

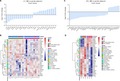

## 1 INTRODUCTION

Brain metastases are the most frequently occurring neurologic complications of cancer, with an estimated incidence of 100 000 to 300 000 patients per year in the USA.^1^ Risk‐years for cancer dissemination to the brain have been increasing, probably because of the availability of upgraded imaging techniques that can assist early diagnosis, and advanced systemic treatments which extend patients' survival. Brain metastases are most common in lung cancer, breast cancer, melanoma, as well as other cancers including colorectal cancer and renal cell cancer.^2^, ^3^, ^4^, ^5^ Metastases can spread to brain parenchyma, leptomeninges, dura, whereas rarely to the pituitary or pineal gland, which are often accompanied by seizures, loss of sensory and/or motor function, cognitive decline, and cranial neuropathies. Brain metastases are usually confirmed by imaging technologies, which can detect lesions of several millimeters in size.^6^

The progressive growth of brain metastases is often associated with advanced primary cancer. The median survival for untreated patients is 1 to 2 months; with the help of conventional radiotherapy and chemotherapy, their lifespan may be extended to 6 months.^7^ Unfortunately, most systemic therapies, such as cytotoxic chemicals and novel targeted agents, have little efficacy against brain metastases, possibly owing to poor penetrability of blood–brain barrier and patient preference for multiple rounds of chemotherapies.^8^, ^9^ However, ipilimumab, a novel immunomodulatory anticytotoxic T lymphocyte‐associated antigen‐4 (CTLA‐4) antibody, has recently shown promising anticancer activity in a group of patients with advanced melanoma and brain metastases.^10^ Although detailed information on the relationship between brain colonization of cancer cells and host immune response is scarce, more and more evidence suggesting that an effective immune response may have great potential in mediating the clinical behavior of brain malignancies. The central nervous system (CNS) is considered as an immune specialized site under a tight regulated network linking microglia, astrocytes, and lymphocytes.^11^ It has been speculated that reactive astrocytes protect cancer cells from chemotherapy by sequestering intracellular calcium or secreting metastasis‐stimulating chemokines.^12^ Reactive glial cells are known to participate in the re‐stimulation of T cells by secreting some chemokines, which is also accompanied by the influx of regulatory T cell (Treg) lymphocytes, thus leading to the silencing of immune response.^13^ Therefore, a profound understanding of the pathogenesis of brain metastases and immune microenvironment is of great importance to support the development of additional active agents for patients with brain metastases.

In this study, we aimed to delineate the immune microenvironment and potential immune response in brain metastases from different primary sites, including the lung, breast, and colon. Briefly, an immune oncology (IO) panel RNA sequencing platform was applied to analyze tissue samples from 25 patients with brain metastasis.

## 2 METHODS

### 2.1 Patients and samples

A total of 25 patients with brain metastasis, including 12 patients with lung cancer, seven with colorectal cancer and six with breast cancer, were admitted to Ruijin Hospital, Shanghai Jiaotong University, School of Medicine. The patients with brain metastasis were diagnosed by systematic examination combined with medical history, such as brain magnetic resonance imaging and chest CT scan, and further confirmed with hematein eosin (HE) staining and specific immunohistochemical staining, which were consistent with the pathological diagnosis of the primary focus. Among them, 13 paired samples of adjacent noncancerous brain tissue (10 from patients with lung cancer and 3 from patients with breast cancer) were collected as controls, whereas the other patients only had brain metastasis tumor tissue. Archived, formalin‐fixed, paraffin‐embedded (FFPE), surgically resected samples of brain metastasis were obtained upon informed consent in accordance with institutional protocols for tissue collection.

### 2.2 RNA extraction, library preparation, and sequencing

The RNA immune oncology (IO) profiling panel is a unique 398‐plex gene expression panel to measure human immune response in solid tumors (Genecast Biotechnology, Beijing, China). The panel was used to determine 395 human genes, which fell into the following categories: immunological function and response to immunotherapy, infiltrating immune cell markers, tumor‐specific antigens, tumor markers and essential signaling pathways, and housekeeping genes. The analytical validation of this panel was reported as previous described.^14^, ^15^ In detail, RNA was extracted from FFPE slides using the truXTRAC FFPE extraction kit (Covaris), according to manufacturer's recommendation. After purification, RNA yield was quantified using Qubit™ RNA HS Assay Kit (Thermo Fisher Scientific). 10 ng RNA was reversely transcribed into cDNA, and targets were amplified with a 398‐plex immune response primer pool targeting 395 genes. Barcode adapters were ligated to FuPa partially digested amplicons and amplified. Purified libraries were quantified via Agilent™ 2100 Bioanalyzer (Agilent), according to manufacturer's instructions. Libraries were pooled in equal molar amounts prior to enrichment and template preparation using the Ion Chef™ system (Thermo Fisher Scientific). 200‐bp sequencing was performed on the Ion S5 530 chip (Thermo Fisher Scientific) to obtain 1‐2 mol/L reads per sample. The absolute read counts of all sequencing samples in the same run were automatically extracted in the in‐house bioinformatics pipeline. The quality control criteria of sequencing data need to meet the following conditions: mapped reads counts ≥200k, valid reads (on target ratio) >50%, and the number of detected housekeeping genes ≥6. Only sequencing data meeting the QC criteria were included in the study.

### 2.3 Gene expression normalization

To make the NGS measurements run to be comparable for evaluation and interpretation, the background subtracted read counts were subsequently normalized to nRPM values as previous described.^15^ Ten housekeeping genes were used as endogenous controls in this panel. Each gene's absolute read count of no template control (NTC) sample was subtracted from that of a clinical sample, to generate a background‐subtracted read count. For any sample, each housekeeping (HK) gene's absolute readout was compared against a predetermined HK reads per million (RPM) profile. The average RPM of a number of replicates of GM12878 cell line samples across different sequencing runs was established as the baseline HK RPM profile, which then gave rise to a fold‐change ratio for each HK gene: Ratio of HK = Absolute read count of HK/ RPM profile of HK. The median value of all HK ratios was then applied as the normalization ratio for the pending sample: Normalization ratio = Median of (all HK ratios). We then calculated the normalized RPM (nRPM) of all genes of the pending sample as: nRPM of (sample S, gene G) = Background‐subtracted read count of (sample S, gene G)/ Normalization ratio of (sample S).

### 2.4 Identification and signature selection of differentially expressed genes

Differentially expressed genes (DEGs) between different types of tumors were identified using the R package, Limma (R‐3.5.1).^16^ Only the genes with |fold‐change| ≥2 and *P* < .05 were identified as DEGs. Significantly enriched signatures were selected using GSEA software. To identify the immune‐related gene clusters associated with the types of primary cancers, we developed the gene signature by measuring the mean level of a class of genes (mean levels of the log2 nRPM values for a subset of genes). The association between the expression of each individual signature and type of primary cancer was then analyzed by logistic regression after adjustment for age and gender.

### 2.5 Statistical analysis

Statistical differences between two independent groups were determined using the Student's *t* test. Logistic regression analysis was conducted with SPSS Statistics 22 (IBM). Heatmap and PCA plot were generated by the R package, pheatmap and ggplot (R‐3.5.1), and the box‐plot was analyzed on Prism 5 (GraphPad Software).

## 3 RESULTS

### 3.1 Patients' characteristics

Brain tissue samples with embedded brain metastases or adjacent noncancerous brain tissue from 25 cancer patients were included in this study (Table 1). Histological diagnoses of the primary cancers included lung cancer (12), breast cancer (6), and colorectal cancer (7). This cohort included 15 male and 10 female patients, with a median age of 62. Among them, 13 samples of matched adjacent noncancerous brain tissues (10 from lung cancer and 3 from breast cancer) were taken as controls. Ten patients received systemic treatment such as chemotherapy, chemo‐radiotherapy, target therapy, whole‐brain radiation, and gamma knife therapy prior to tissue sampling of the metastatic lesions. The information on the estrogen receptor (ER), progesterone receptor (PR), and HER‐2 status of the six breast cancer cases was included in Table 1. Among them, five of six were ER(−), PR(−), HER‐2(+), whereas another one was ER(+), PR(+), HER‐2(+).

**Table 1:** Patients' characteristics

Patient	Primary cancer type	Age	Gender	Brain metastases tumor tissue	Noncancerous adjacent brain tissue	Systemic treatment prior to BM resection	ER, PR, HER‐2 status
1	Lung cancer	64	Male	+	+	No	
2	Lung cancer	61	Male	+	+	No	
3	Lung cancer	74	Male	+	+	No	
4	Lung cancer	80	Male	+	+	No	
5	Lung cancer	64	Male	+		Chemoradiotherapy	
6	Lung cancer	62	Male	+	+	No	
7	Lung cancer	66	Female	+	+	Target therapy	
8	Lung cancer	57	Female	+		Chemoradiotherapy + target therapy	
9	Lung cancer	49	Male	+	+	No	
10	Lung cancer	53	Male	+	+	No	
11	Lung cancer	72	Male	+	+	No	
12	Lung cancer	49	Male	+	+	No	
13	Breast cancer	69	Female	+	+	No	ER (−), PR (−), Her‐2 (2+)
14	Breast cancer	53	Female	+	+	Chemoradiotherapy	ER (−), PR (−), Her‐2 (1+)
15	Breast cancer	52	Female	+	+	Chemoradiotherapy	ER (−), PR (−), Her‐2 (3+)
16	Breast cancer	64	Female	+		Gamma knife therapy	ER (−), PR (−), Her‐2 (3+)
17	Breast cancer	39	Female	+		Chemotherapy	ER (−), PR (−), Her‐2 (1+)
18	Breast cancer	54	Female	+		No	ER (+), PR (+), Her‐2 (2+)
19	Colorectal cancer	77	Female	+		No	
20	Colorectal cancer	29	Male	+		Chemotherapy	
21	Colorectal cancer	67	Male	+		No	
22	Colorectal cancer	68	Male	+		Chemotherapy	
23	Colorectal cancer	50	Male	+		Whole brain radiation + chemotherapy	
24	Colorectal cancer	60	Male	+		No	
25	Colorectal cancer	78	Female	+		No	

### 3.2 Immunological characteristics of brain metastases and surrounding microenvironments

**Figure 1:**
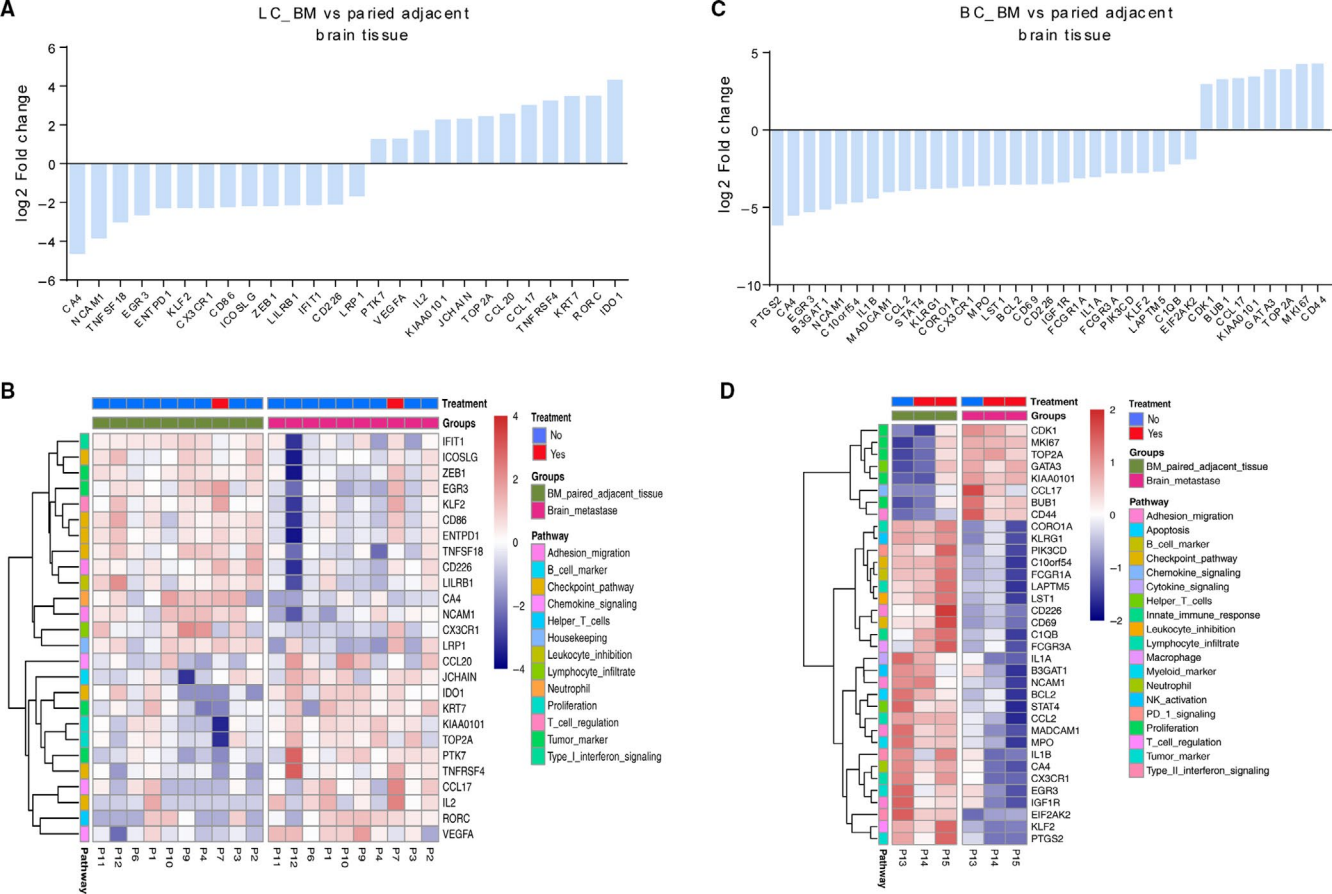
RNA profiling of brain metastases and paired adjacent noncancerous brain tissue by RNA immune oncology profiling panel. A and B, Differentially expressed genes (DEGs) in 10 paired lung cancer arising brain metastases and adjacent noncancerous brain tissue. C and D, DEGs in three paired breast cancer arising brain metastases and adjacent noncancerous brain tissue. The expression levels of immune related genes were deemed significantly expressed if the median ratio was ≥2 and the *P* value was <.05. Patient who received systemic treatment prior to tissue sampling of the metastatic lesions was marked yes (horizontal, red), otherwise no (horizontal, blue). BC, breast cancer; BM, brain metastases; LC, lung cancer

To characterize the immune microenvironment in and around brain metastases, we employed an RNA IO profiling panel which simultaneously quantified 395 immune‐related genes in a single reaction. A brief summary of 395‐gene RNA IO panel information, the quality of sequencing data, and the gene expression profile of analyzed samples were described in supplemental Table S1, S2, and S3, respectively. First, by comparing the brain metastases and paired adjacent noncancerous brain tissue in 10 patients who suffered from lung cancer, we found that 12 genes were upregulated and 14 genes were downregulated in brain metastases, among which indoleamine 23‐dioxygenase 1 (IDO1), RAR‐related orphan receptor C (RORC), carbonic anhydrase 4 (CA4), and neural cell adhesion molecule 1 (NCAM1) were the top‐ranked upregulated or downregulated genes (Figure 1A,B). Second, we assessed three brain metastases and paired adjacent noncancerous brain tissue from three patients with breast cancer, and observed 35 differentially expressed genes (DEGs) including eight upregulated and 27 downregulated genes in the brain metastases compared with the controls (Figure 1C,D). Since the immune microenvironment is dynamic and may be impacted by systemic therapies, we analyzed whether these DEGs we found were influenced by systemic therapies prior to tissue sampling of the metastatic lesions, and noticed that systemic treatment was not relevant to our results (Figure 1B,D).

**Figure 2:**
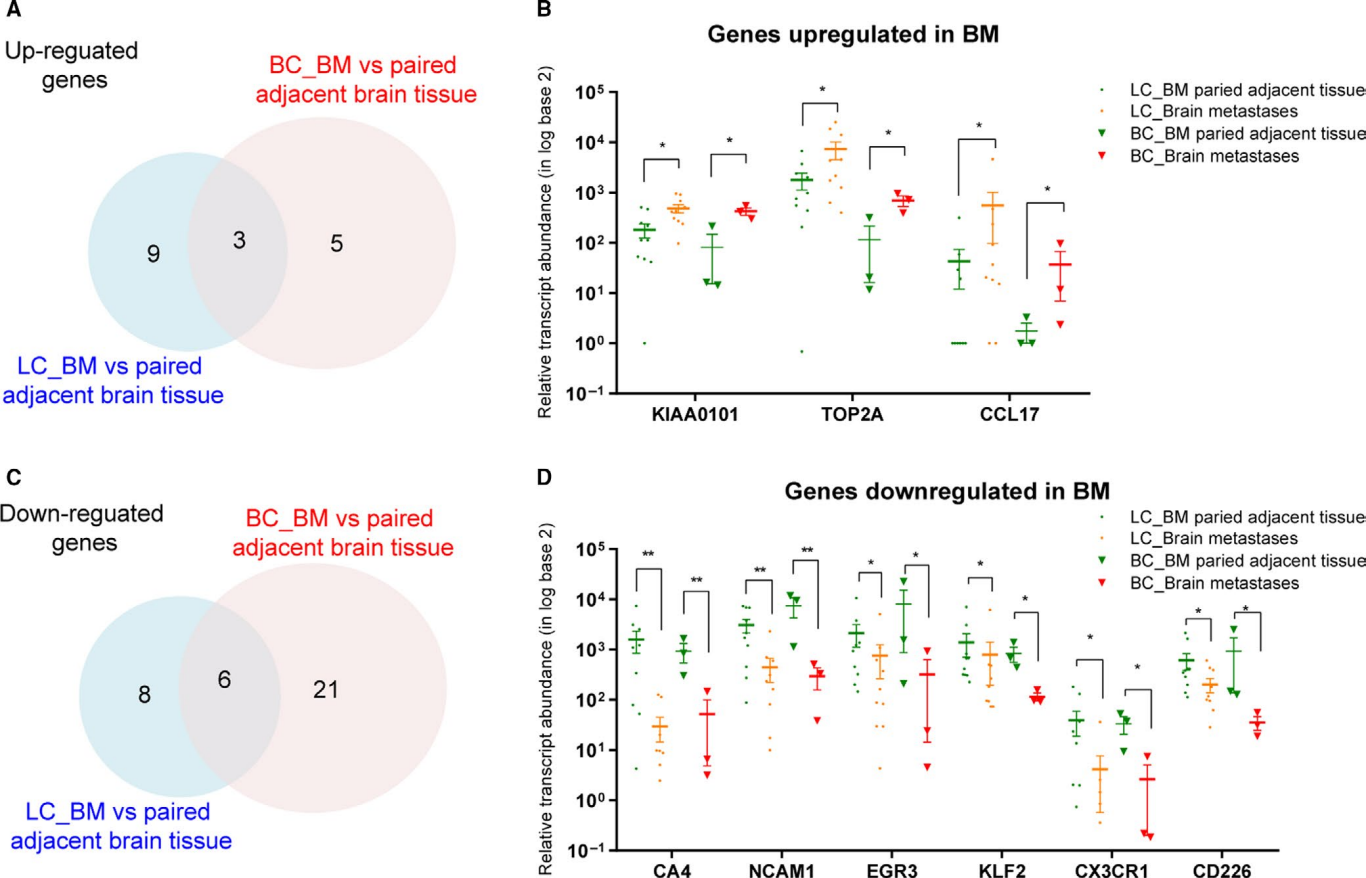
Identification of co‐regulated genes differentially expressed in different cancer types. A, Venn diagram indicates the overlap of overexpressed genes identified in paired brain metastases and control tissue from patients with lung cancer or breast cancer. B, Relative transcript abundance of co‐upregulated genes in each sample was shown as nRPM values. C, Venn diagram indicates the overlap of downregulated genes identified in paired brain metastases and control tissue from patients with lung cancer or breast cancer. D, Relative transcript abundance of co‐downregulated genes in each sample was shown as nRPM values. *, *P* < .05; **, *P* < .01. BC, breast cancer; BM, brain metastasis; LC, lung cancer

Moreover, to identify common differentially expressed genes from the two cancer types, a cross comparison was performed between the two subsets of genes, as demonstrated in Venn Diagram (Figure 2A,C). Generally, we obtained three co‐upregulated genes [KIAA0101, topoisomerase DNA II alpha (TOP2A), and C‐C motif chemokine ligand 17 (CCL17)] and six co‐downregulated genes [carbonic anhydrase 4 (CA4), neural cell adhesion molecule 1 (NCAM1), early growth response 3 (EGR3), Kruppel like factor 2 (KLF2), C‐X3‐C motif chemokine receptor 1 (CX3CR1), and CD226] in the two types of brain metastases (Figure 2B,D).

### 3.3 Immunological landscape of brain metastases from different cancer types

**Figure 3:**
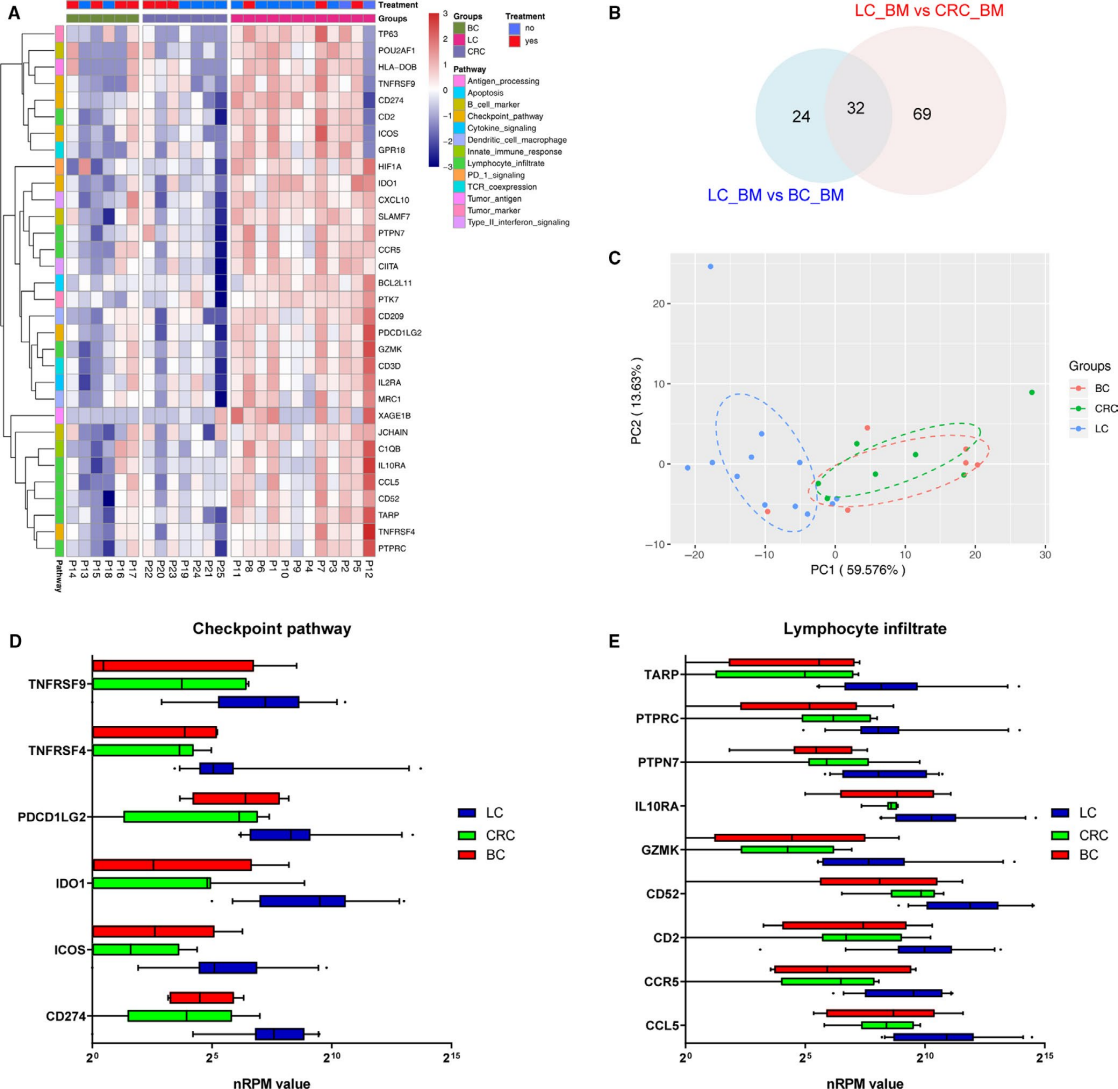
Immunological landscape of brain metastases from lung, breast and colorectal cancer patients. A, Heatmap of RNA expression profiles in brain metastases from lung (horizontal, purple), breast (horizontal, green), and colorectal cancer patients (horizontal, blue). B, Venn diagram indicates the overlap of DEGs identified in lung cancer cases when compared with breast cancer or colorectal cancer patients. C, PCA plot showed the difference across cancer types. D and E, Boxplot of the nRPM values in genes belonging to the checkpoint pathway or lymphocyte infiltration pathways. Patient who received systemic treatment prior to tissue sampling of the metastatic lesions was marked yes (horizontal, red), otherwise no (horizontal, blue). BC, breast cancer; BM, brain metastases; CRC, colorectal cancer; LC, lung cancer; PCA, principal component analysis

The incidence of brain metastasis is highest in lung cancer, followed by breast cancer, melanoma, renal cell cancer, gastrointestinal cancer, other solid tumors, and metastases from unknown primary cancers.^2^ Herein, we collected 25 brain metastases from 25 patients, including 12 with lung cancer, seven with colorectal cancer, and six with breast cancer. All these metastases were subjected to RNA IO sequencing, with nRPM values yielded for each of the 395 genes in the panel. To profile immune‐related genes in brain metastases from the three primary cancer types, we compared differentially expressed genes in brain metastases originating from lung cancer and other cancer types. Compared with breast cancer, there were 56 differentially expressed genes in brain metastases of lung cancer, 52 of which were upregulated in lung cancer. Similarly, there were 101 differentially expressed genes in brain metastases of lung cancer compared with that of colorectal cancer, 94 of which were upregulated genes in lung cancer. As shown in Figure 3A,B, there were 32 overlapped differentially expressed genes in brain metastases from lung cancer compared with those from breast and colorectal cancers, all of which were overexpressed in lung cancer. These upregulated genes could be classified into the following: checkpoint pathways, lymphocyte infiltration, TCR‐coexpression, tumor antigen, and tumor markers. A PCA plot indicated that these overlapped differentially expressed genes might distinguish lung cancer from breast cancer and colorectal cancer (Figure 3C).

Interestingly, PD‐L1/CD274, PD‐L2/PDCDLG2, and IDO1, which belong to the checkpoint pathways, were highly expressed in the brain metastases of lung cancer (Figure 3D). It has been reported that the interaction between programmed death‐1 (PD‐1) and its ligands, PD‐L1 and PD‐L2, contributes to the inhibition of T‐cell function and immune evasion of tumor cells.^17^, ^18^ IDO1 has also emerged as an intriguing target implicated in tumoral immune escape, and may act as a nodal pathogenic driver gene for lung cancer development and metastasis.^19^ On the other hand, a number of genes associated with lymphocyte infiltration also increased in the brain metastases of lung cancer, including protein tyrosine phosphatase nonreceptor type 7 (PTPN7), interleukin 10 receptor subunit alpha (IL10RA), granzyme K (GZMK), CD52 molecule (CD52), CD2 molecule (CD2), C‐C motif chemokine receptor 5 gene pseudogene (CCR5), and C‐C motif chemokine ligand 5 (CCL5) (Figure 3E). Taken together, our findings indicated that brain metastases from lung cancer might benefit from immune checkpoint therapy.

### 3.4 Distinct immune signatures distinguishing brain metastases originating from lung and breast cancers

A large proportion of cancer patients with intracranial metastases had synchronous extracerebral metastases. Some patients presented with unknown primary cancer diagnosis, accounting for 2%‐14% of all patients with brain metastases based on different institutions and cancer centers.^20^, ^21^, ^22^, ^23^ Gene expression profiling of various cancers has facilitated the identification of molecular signatures and the development of RNA biomarkers for diagnosis and prognosis. Herein, by presenting this classification, we aimed to select specific immune RNA signatures that may help distinguish brain metastases from lung and breast, which are the top‐ranked primary sites of brain metastases.

**Figure 4:**
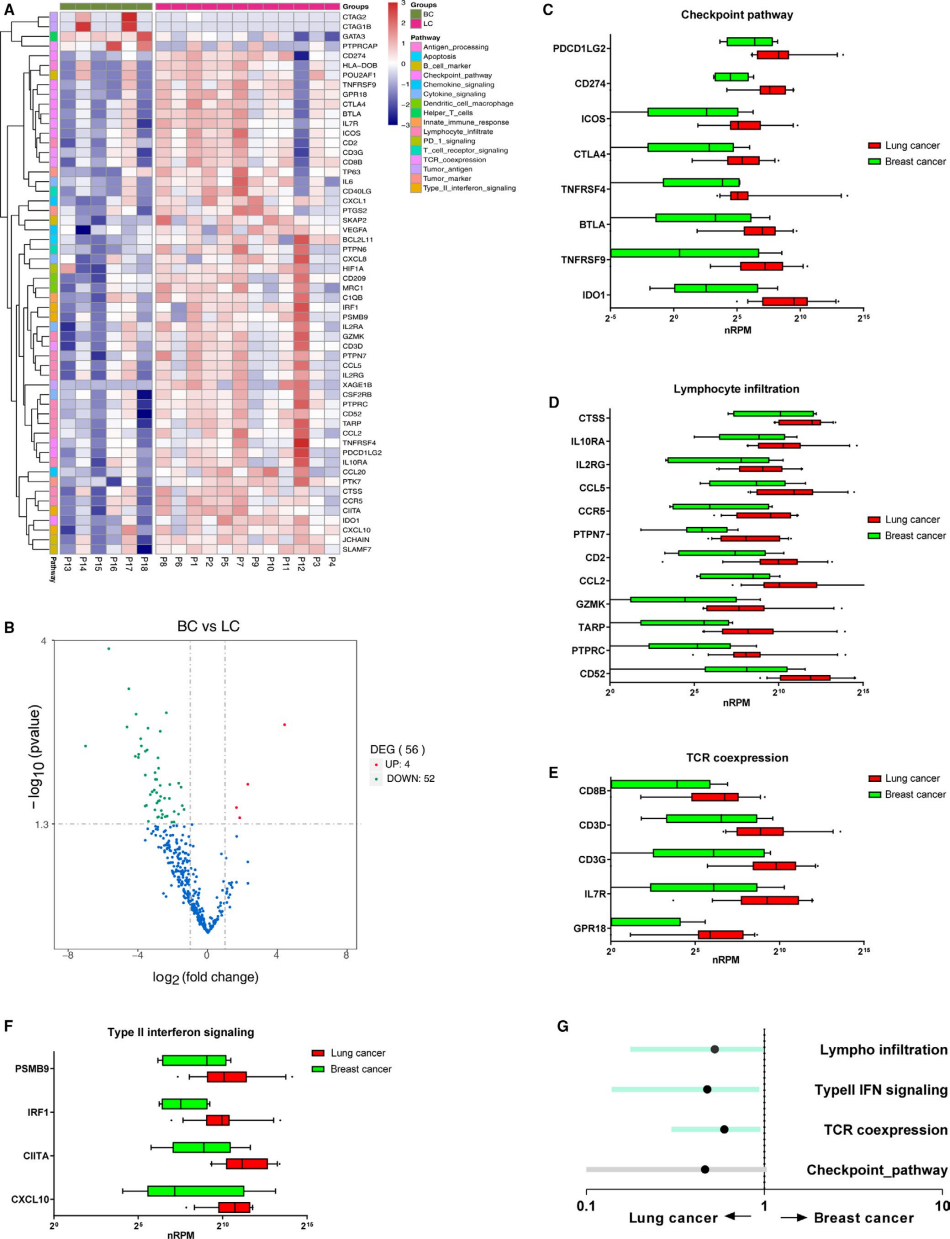
Distinct immune signatures exist between brain metastases from lung and breast cancers. A, Heatmap of RNA expression profiles in brain metastases from lung (horizontal, purple) and breast (horizontal, green) cancers. B, Volcano plot diagram indicates the upregulated and downregulated DEGs identified in lung cancer compared with breast cancer. C‐F, Boxplot of nRPM values in genes belonging to the checkpoint pathway (C), lymphocyte infiltration pathways (D), TCR‐coexpresssion (E), and type II interferon signaling (F). G, We analyzed the association between the expression of each individual signature and type of primary cancer using logistic regression analysis after adjustment for age and gender. Statistically significant signatures are shown in blue (*P* < .05). BC, breast cancer; IFN, interferon; LC, lung cancer

Briefly, 52 differentially expressed genes were obtained by comparing brain metastases originating from lung and breast (Figure 4), which can be divided into four classes: checkpoint pathways, lymphocyte infiltration, TCR‐coexpression, and type II interferon signaling. Except for the increase in gene clusters, which were mainly attributed to intracranial metastases from lung cancer (Figure 3 and Figure 4A‐D), we also observed a series of downregulated genes in brain metastases from breast cancer (Figure 4E,F), including TCR coexpression‐related genes [G‐protein‐coupled receptor 18 (GPR18), interleukin 7 receptor (IL7R), CD3‐gamma molecule (CD3G), CD3‐delta molecule (CD3D), and CD8b molecule (CD8B)] and genes belonging to type II interferon signaling pathway [C‐X‐C motif chemokine ligand 10 (CXCL10), class II major histocompatibility complex transactivator (CIITA), interferon regulatory factor 1 (IRF1), and proteasome subunit beta 9 (PSMB9)]. To identify immune‐related biological processes associated with the primary sites of brain metastases, we evaluated the association between the expression of each individual signature summarized in Figure 4C‐F and the primary cancer types (Figure 4G). Among different biomarkers, three signatures (lymphocyte infiltration, TCR coexpression, and type II interferon signaling) were found to be significantly related with the occurrence of lung cancer‐derived brain metastases. This observation suggested that brain metastases originating from lung cancer might have a more favorable outcome after immunotherapy than those from breast or colorectal cancer, due to higher expression of genes belonging to the PD‐1/PD‐L1 axis, tumor‐infiltrating markers, and interferon signatures.

## 4 DISCUSSION

Molecular factors that contribute to brain metastases, such as genes involved in cell metabolism, extravasation, and adhesion, as well as multiple cellular signaling pathways, have been extensively explored. Intriguingly, the role of the unique brain microenvironment has attracted considerable attention in recent years, especially the “seed and soil” hypothesis, which substantiates the interaction between the tumor cell (the “seed”) and the congenial microenvironment (the “soil”).^2^ The outcome of metastases largely depends on the various interactions between metastatic cells and surrounding homeostatic mechanisms that the cancer cells exploit for their survival and proliferation. Of note, the novel immunomodulatory therapy has recently received substantial attention for its promising antitumor activity in patients with brain metastases.^10^ Therefore, a comprehensive evaluation of the immune characteristics in various malignant brain metastases from different cancer types would be of great essentiality.

We first characterized the immune microenvironment in and surrounding the brain metastases (10 paired tissue samples from patients with lung cancer and three from breast cancer) using a RNA immune oncology (RNA IO) sequencing platform. Notably, the three co‐upregulated genes (KIAA0101; TOP2A, and CCL17) in brain metastases originating from primary lung cancer and breast cancer were mainly “tumor markers” (Figure 2A,B), representing malignant properties of cancer cells that accelerated DNA replication and mediated mitogenic stimulation.^24^, ^25^ More importantly, CCL17 is a C‐C chemokine that selectively attracts Th2‐type lymphocytes and may contribute to the immunosuppression triggered by tumor‐associated neutrophils (TANs) in the tumor microenvironment.^26^ On the other hand, our data revealed that downregulated essential genes (CA4, NCAM1, EGR3, KLF2, CX3CR1, and CD226) in both types of brain metastases mainly fell into two functional subgroups: cell adhesion and lymphocyte regulation, indicating that increased cell motility and a malfunction of the cellular immune response in the microenvironment might collectively result in brain colonization of cancer cells. However, the samples are limited, especially only three paired samples for breast cancer. More samples are needed to draw robust conclusions in the future study. Besides, in‐depth functional and biochemical study is needed to elucidate the exact molecular mechanism underlying the highly metastatic activity of lung cancer and breast cancer in the brain.

Next, we profiled immune‐related genes in brain metastases across three cancer types including lung, breast, and colorectal cancers. Surprisingly, immune checkpoint molecules PD‐L1, PD‐L2, and IDO1 were found to be highly expressed in brain metastases from lung cancer. The PD‐1 and PD‐L2 receptors are immune checkpoint inhibitors, and the binding of PD‐1 to its ligands (PD‐L1 and PD‐L2) on tumor cells has been reported to suppress T cells through a negative feedback loop, leading to evasion of the immune response.^27^, ^28^ Recently, three agents, namely, nivolumab (Opdivo [Bristol‐Myers Squibb]), pembrolizumab (Keytruda [Merck & Co., Inc.]), and atezolizumab (Tecentriq [Genentech/Roche]), which target the PD‐1/PD‐L1 axis, have been approved by the US Food and Drug Administration (FDA) for patients with advanced non‐small‐cell lung cancer (NSCLC) after failure of first‐line therapy.^29^ Specifically, in a single‐institution, two‐cohort, phase II trial, 6 of 18 patients with brain metastasis originating from NSCLC had a 33% response rate to pembrolizumab, demonstrating an acceptable safety profile for its activity in brain metastases from NSCLC.^30^ Hence, it would be of great interest to explore the efficacy and safety of these agents in patients with untreated brain metastases.

A retrospective study of 342 patients diagnosed with brain metastases revealed that over 30% had an undefined primary site. Further diagnostic workup of these patients showed that the primary sites were the lung and other organs in 60% and 14% of the cases, respectively. In 26% of the cases, however, no primary site was determined despite thorough diagnostic workup.^31^ Therefore, the RNA expression profiling data, as presented in Figure 4, had provided a group of molecular signatures for potential diagnosis of brain metastases from unknown primary sites (Figure 4). However, more samples and a validation cohort are required to construct a well‐established RNA signature.

## 5 CONCLUSIONS

The immunological microenvironments of brain metastases from different cancer types have revealed distinct properties. Further studies would shed light on whether strategies designed to activate specific immune responses are functional to exert therapeutic effect against brain metastases, especially those from lung cancer.

## CONFLICT OF INTEREST

The authors declare that they have no competing interests.

## AUTHOR CONTRIBUTIONS

This study was conceived by JZ, HZ, and HZ designed the study; JJ, LW, and FY performed the experiments; JJ, JJ, XL, WY, JW, MS, HY, YM, and XS analyzed and interpreted the data; ZZ, HZ, and JZ reviewed the manuscript; and HZ and JZ wrote the manuscript with comments from all authors. All authors read and approved the final manuscript.
